# 25-Hydroxyvitamin D_3_ Deficiency Independently Predicts Cognitive Impairment in Patients with Systemic Lupus Erythematosus

**DOI:** 10.1371/journal.pone.0144149

**Published:** 2015-12-04

**Authors:** Sen Hee Tay, Chung Shun Ho, Roger Chun-Man Ho, Anselm Mak

**Affiliations:** 1 Department of Medicine, Yong Loo Lin School of Medicine, National University of Singapore, Singapore; 2 Division of Rheumatology, Department of Medicine, National University Hospital, National University Health System, Singapore; 3 Biomedical Mass Spectrometry Unit, Department of Chemical Pathology, The Chinese University of Hong Kong, Prince of Wales Hospital, Hong Kong SAR, China; 4 Department of Psychological Medicine, Yong Loo Lin School of Medicine, National University of Singapore, Singapore; Instituto Nacional de Ciencias Medicas y Nutricion Salvador Zubiran, MEXICO

## Abstract

**Objectives:**

Cognitive dysfunction has been reported in 20–80% of SLE patients. Converging evidence has indicated the importance of vitamin D as a neuroimmunomodulator for cognitive function. In this study, we evaluated the relationship between vitamin D and cognitive dysfunction.

**Methods:**

Consecutive age- and gender-matched SLE patients and healthy controls (HCs) were administered Automated Neuropsychological Assessment Metrics in this cross-sectional study. The primary outcome was the total throughput score (TTS). Anxiety and depression were measured using the Hospital Anxiety and Depression Scale (HADS). Levels of 25-hydroxyvitamin D [25(OH)D_3_ and total 25(OH)D] were measured using Liquid Chromatography-Tandem Mass Spectrometry.

**Results:**

In total, 61 SLE patients and 61 HCs were studied. SLE patients scored significantly lower than HCs in the TTS (p = 0.004). There were no statistically significant differences in 25(OH)D_3_ levels, total 25(OH)D levels and total 25(OH)D deficiency between SLE patients and HCs. However, more SLE patients had 25(OH)D_3_ deficiency compared to HCs [12 (19.7%) versus 2 (3.3%), p = 0.003]. Deficiency of 25(OH)D_3_ (β = -63.667, SE = 27.456, p = 0.025), but not other vitamin D variables, independently predicted worse TTS after adjusting for age, education, gender, ethnicity, HADS-Total, duration of SLE, SELENA-SLEDAI, SLICC/ACR Damage Index and cumulative steroid dose in SLE patients. Age (β = -4.261, SE = 0.866, p < 0.001) was the only predictor of TTS after adjusting for education, gender, ethnicity, HADS-Total, vitamin D levels or status in HCs.

**Conclusions:**

Deficiency of 25(OH)D_3_, a potentially modifiable risk factor, independently predicted cognitive impairment in SLE patients.

## Introduction

Systemic lupus erythematosus (SLE) is a chronic, systemic, relapsing-remitting autoimmune disease of unknown etiology with heterogeneous manifestations, including diverse neuropsychiatric (NP) manifestations [1]. Neurologic and psychiatric syndromes, collectively referred to as neuropsychiatric systemic lupus erythematosus (NPSLE), occur frequently in patients with SLE [2]. Approximately one-third of NP events are directly attributable to SLE, although the attribution rates vary between individual manifestations [2]. Over the past few decades, as the longevity of SLE patients has increased, NPSLE has been identified as one of the important factors negatively affecting of survival of SLE patients over the past 50 years [1, 3]. Among the 19 NPSLE syndromes identified by the American College of Rheumatology (ACR) Ad Hoc Committee on Neuropsychiatric Lupus Nomenclature, cognitive dysfunction is the most prevalent manifestation of NPSLE and has been reported in up to 20–80% of SLE patients [3, 4]. The variability in the frequency of cognitive dysfunction is due to several factors, including bias in selection of patients for study and operational decisions regarding the definition of cognitive dysfunction [5]. Cognitive impairment scores as an item in the Systemic Lupus International Collaborating Clinics/American College of Rheumatology (SLICC/ACR) Damage Index [6]. Accrual of such damage from cognitive dysfunction translates to increased negative impact on health-related quality of life and unemployment rates [7, 8].

Identifying nutritional factors that may mitigate cognitive dysfunction and help preserve higher-level cognitive abilities has significant economic and public health benefits [9]. Epidemiological studies within the general population have demonstrated that vitamin D deficiency is a potential risk factor for cognitive impairment [10, 11]. It has also become recently apparent that vitamin D deficiency contributes to the disease activity and morbidity of SLE [12]. Vitamin D is available in 2 distinct forms, vitamin D_2_ (ergocalciferol) and vitamin D_3_ (cholecalciferol) [13]. The 2011 Institute of Medicine report concluded that serum 25(OH)D is the most useful marker for vitamin D nutriture, without distinguishing between 25(OH)D_2_ and 25(OH)D_3_ forms [14]. However, as vitamin D_3_ is more effective than vitamin D_2_ in raising 25(OH)D concentrations and experimental evidence has revealed that binding of 1,25(OH)_2_D_3_ to its cognate nuclear vitamin D receptor (VDR) represents an important molecular event, we hypothesize that measurement of 25(OH)D_3_ represents a more accurate estimation of *in vivo* vitamin D status and may thus affect cognitive function in SLE patients [13]. To our knowledge, the relationship between vitamin D and cognitive function in patients with SLE has never been addressed. We undertook this study to evaluate the relationship between 25(OH)D_3_, traditional neuropsychological and SLE-specific risk factors and cognitive dysfunction in a multiethnic Asian population living close to the equator.

## Patients and Methods

### Subjects

Adult SLE patients attending the outpatient clinics of National University Hospital (NUH), Singapore, between 2011 and 2014 who fulfilled at least 4 of the ACR 1997 revised classification criteria were invited to participate in this study [15]. Objectively documented cognitive dysfunction or other NP events were not required for inclusion. Exclusion criteria included the following: (i) patients with known intellectual disability or previous stroke with resultant neuromuscular dysfunction; (ii) subjects who were pregnant; (iii) subjects less than 21 years of age and (iv) subjects who had active infections at the time of recruitment. Community-derived healthy controls (HCs) were recruited using poster advertisement in the hospital or were the nursing staff of NUH. The HCs were selected for the absence of any acute or chronic psychiatric, neurologic or medical conditions. Those who required medications which could alter cognitive function were excluded. All subjects had completed at least primary school education with adequate English fluency. The study was approved by NHG Domain Specific Review Board E (reference code: 2011/01764) and was carried out in accordance with the principles of the Declaration of Helsinki. All subjects gave written informed consent prior to study inclusion.

### Study design

The study was cross-sectional in design. Consecutive SLE patients and community-derived HCs matched by age (± 5years) and gender were recruited.

### SLE-related disease characteristics

Demographic and relevant clinical data were extracted from the medical records of SLE patients. NP events were compiled by the study rheumatologists (S.H. Tay and A. Mak). Global SLE disease activity and SLE-related disease damage were assessed using the Safety of Estrogens in Lupus Erythematosus National Assessment (SELENA)-modified SLE Disease Activity Index (SLEDAI) and the SLICC/ACR Damage Index, respectively [6, 16].

### Psychopathology questionnaire

The Hospital Anxiety and Depression Scale (HADS) was used to assess self-reported symptoms of anxiety and depression. It is a 14-item multiple-choice questionnaire which comprises two 7-item subscales for measuring anxiety (HADS-Anxiety) and depression (HADS-Depression). Scores range from 0–21 for each subscale. A cut-off score of ≥ 8 on either subscale is used to define clinical anxiety and depression [17]. Summing scores across both subscales generates a total psychopathology score (HADS-Total).

### Neuropsychological test and definition of cognitive dysfunction

Cognitive function was evaluated using Automated Neuropsychological Assessment Metrics (ANAM), version 4.3 (Vista LifeSciences Inc, Denver, CO, US). The ANAM is a self-administered computer battery of tests to assess neurocognitive efficiency and it is less sensitive to confounding effects of education, English language proficiency and ethnic differences than traditional neuropsychological testing [18]. ANAM tests selected for this study included simple reaction time (neuromuscular response efficiency, 40 trials) and 8 tests: (i) code substitution-learning (learning and recall, 72 trials); (ii) code substitution-immediate (learning and recall, 36 trials); (iii) code substitution-delayed (learning and recall, 36 trials); (iv) spatial processing (visual perception and mental rotation, 20 trials); (v) matching to sample (short-term memory, attention and visual-spatial discrimination, 20 trials); (vi) running memory continuous performance test (CPT) (sustained attention, 80 trials); (vii) mathematical processing (working memory, 20 trials) and (viii) memory search (working memory, 40 trials). Each ANAM test generates a “throughput” (TP) measure, which is sensitive to cognitive performance, incorporates speed and accuracy in one variable and more closely conforms to a normal distribution than other ANAM measures [19]. The primary outcome of our study was the total throughput score (TTS), which is the total of the throughput scores for each of the 8 ANAM tests [20]. Cognitive dysfunction was defined as TTS < 1.5 SD below the mean of the HCs [20].

### Assay of 25(OH)D_3_ and definition of vitamin D deficiency

Quantitative determination of serum 25(OH)D_3_ was performed at an external laboratory (Prince of Wales Hospital, Hong Kong SAR, China) using liquid chromatography-tandem mass spectrometry (LC-MS/MS) as the candidate reference method to measure both 25(OH)D_3_ and total 25(OH)D [25(OH)D_2_ + 25(OH)D_3_] [21]. Processed serum was stored at -80°C until retrieval for analyses. Total 25(OH)D was also measured using the Roche Elecsys vitamin D total assay immediately after collection at the local laboratory of NUH. Vitamin D status was operationally defined as deficiency < 10 ng/mL, insufficient 10–29 ng/mL and sufficient ≥ 30 ng/mL as per the clinical laboratory reference ranges of NUH.

### Study procedures

For each subject, HADS, ANAM, and if applicable, SELENA-SLEDAI and SLICC/ACR Damage Index were assessed on the day when serum samples were collected.

### Statistical methods

As most of the published normative data are for young men (because ANAM was developed by the US Department of Defense) and since SLE patients could not be their own controls in a cross-sectional study; therefore, a control group was required to assess (i) prevalence of cognitive dysfunction and (ii) the association of vitamin D deficiency with cognitive impairment in SLE patients [22, 23]. 25(OH)D_3_ and total 25(OH)D levels were examined as continuous variables and also as deficient, insufficient and sufficient categorical variables. Multiple linear regression was used to identify independent predictors for TTS and to test for group differences in the TTS while adjusting for potential confounders. In all models, we controlled for baseline confounders including age, education, gender, ethnicity and HADS-Total. In the fully adjusted model, we adjusted for SLE-specific variables that have been identified as potential confounders in studies of cognition: duration of SLE, SELENA-SLEDAI, SLICC/ACR Damage Index and cumulative steroid dose. To ascertain the validity of the regression equations, only independent variables of tolerance > 0.4 were accepted into the final regression model. Statistical significance was defined as a two-tailed p value of < 0.05. All statistical analyses were performed with SPSS, version 23.0 (IBM Corp, Armonk, NY, US).

## Results

### Characteristics of SLE patients and healthy controls

In total, 61 SLE patients and 61 HCs were studied. The SLE patients and HCs were comparable in terms of age, gender and body mass index (Table 1). However, there were statistically significant differences in ethnicity and duration of education [SLE patients: 12.0 years (Q1; Q3 10.0; 15.0) versus HCs: 16.0 years (Q1; Q3 14.0; 18.0), p < 0.001]. SLE patients had the following characteristics: 83.6% female; median age 36.0 (Q1; Q3 26.0; 48.5); SLE duration 6.0 years (Q1; Q3 0.0; 12.0); SELENA-SLEDAI 4.0 (Q1; Q3 2.0; 5.0) and SLICC/ACR Damage Index 0.0 (Q1; Q3 0.0; 1.0) (Tables 1 and 2). Fifty-six (91.8%) of the 61 SLE patients were taking prednisolone [median dosage 7.5 mg/day (Q1; Q3 4.5; 16.3)] with a cumulative prednisolone dose of 15.9 gm (Q1; Q3 7.4; 26.9) (Table 2). Twelve (19.7%) of the 61 SLE patients had at least 1 NP event but no patient had documented cognitive dysfunction (Table 2). Psychopathology parameters which included HADS-Anxiety, HADS-Depression and HADS-Total were significantly higher in SLE patients than those of HCs [HADS-Anxiety: 8.0 (Q1; Q3 4.5; 9.0) versus 5.0 (Q1; Q3 2.5; 8.0), p = 0.001; HADS-Depression: 3.0 (Q1; Q3 2.0; 6.0) versus 2.0 (Q1; Q3 1.0; 3.0), p < 0.001; HADS-Total: 11.0 (Q1; Q3 7.5; 15.0) versus 7.0 (Q1; Q3 3.0; 10.5), p < 0.001] (Table 1). Significantly more SLE patients had clinical anxiety (50.8% versus 26.2%, p = 0.005) and depression (14.8% versus 1.6%, p = 0.008) than HCs (Table 1).

**Table 1:** Demographics, clinical and psychological characteristics of SLE patients versus healthy controls. Data are no./no. assessed (%) or median (interquartile range).

	SLE patients, n = 61	Healthy controls, n = 61	p
Age (years)	36.0 (26.0–48.5)	29.0 (25.0–40.5)	0.091
Gender (female)	51 (83.6)	51 (83.6)	1.000
Ethnicity			0.000
Chinese	33 (54.1)	49 (80.3)	
Malay	16 (26.2)	2 (3.3)	
Indian	8 (13.1)	1 (1.6)	
Others*	4 (6.6)	9 (14.8)	
Education (years)	12.0 (10.0–15.0)	16.0 (14.0–18.0)	0.000
Body mass index (kg/m^2^)	23.0 (18.9–26.0)	22.3 (19.7–25.2)	0.834
Menopause	12/51 (23.5)	5/51 (9.8)	0.062
Current smoking	7 (11.5)	1 (1.6)	0.025
Diabetes mellitus	2 (3.3)	2 (3.3)	1.000
Hypertension	22 (36.1)	2 (3.3)	0.000
Hypercholesterolemia	19 (31.1)	7 (11.5)	0.008
LDL cholesterol (mmol/L)	2.45 (2.14–2.94)	2.77 (2.41–3.35)	0.056
HDL cholesterol (mmol/L)	1.43 (1.04–1.69)	1.54 (1.34–1.98)	0.065
Total cholesterol (mmol/L)	4.63 (4.00–5.25)	4.87 (4.33–5.35)	0.188
Triglyceride level (mmol/L)	1.12 (0.79–1.58)	0.83 (0.65–1.15)	0.015
Total/HDL cholesterol ratio	3.29 (2.52–4.51)	3.00 (2.48–3.64)	0.129
History of cardiovascular disease	9 (14.8)	0 (0.0)	0.002
History of stroke**	4 (6.6)	0 (0.0)	0.040
History of myocardial infarction	4 (6.6)	0 (0.0)	0.040
Total 25(OH)D (ng/mL)^†^	22.7 (16.1–30.6)	19.7 (15.0–25.8)	0.112
Total 25(OH)D (ng/mL)^††^	22.8 (17.6–29.0)	19.6 (16.0–24.8)	0.078
25(OH)D_3_ (ng/mL)^††^	20.0 (12.0–24.8)	19.6 (16.0–24.8)	0.456
Vitamin D_2_ or D_3_ supplementation	57 (93.4)	12 (19.7)	0.000
Vitamin D_2_ supplementation	8 (13.1)	0 (0.0)	0.000
Total 25(OH)D deficiency^††^	5 (8.2)	2 (3.3)	0.224
25(OH)D_3_ deficiency^††^	12 (19.7)	2 (3.3)	0.003
HADS-Total	11.0 (7.5–15.0)	7.0 (3.0–10.5)	0.000
HADS-Anxiety (0–21)	8.0 (4.5–9.0)	5.0 (2.5–8.0)	0.001
Anxiety			0.005
≥8	31 (50.8)	16 (26.2)	
<8	30 (49.2)	45 (73.8)	
HADS-Depression (0–21)	3.0 (2.0–6.0)	2.0 (1.0–3.0)	0.000
Depression			0.008
≥8	9 (14.8)	1 (1.6)	
<8	52 (85.2)	60 (98.4)	

* All were Burmese or Filipinos

** All 4 SLE patients with stroke did not have impaired mobility

† Measured using Roche Elecsys vitamin D total assay

^††^ Measured using LC–MS/MS.

**Table 2:** Cumulative clinical features of SLE patients at time of study recruitment. Data are no./no. assessed (%) or median (interquartile range).

Age at diagnosis of SLE (years)	28.0 (19.0–36.0)
Duration of SLE (years)	6.0 (0.0–12.0)
SELENA-SLEDAI	4.0 (2.0–5.0)
SLICC/ACR Damage Index	0.0 (0.0–1.0)
Positive anti-dsDNA	31 (50.8)
Anti-dsDNA (IU)	42.0 (11.0–188.5)
Low C3	31 (50.8)
Low C4	19 (31.1)
Positive antiphospholipid antibodies	20 (32.8)
Positive lupus anticoagulant	9 (14.8)
ACR classification criteria	
Malar rash	21 (34.4)
Discoid rash	4 (6.6)
Photosensitivity	14 (23.0)
Oral ulcers	11 (18.0)
Arthritis	38 (62.3)
Serositis	19 (31.1)
Renal disorder	28 (45.9)
Neurologic disorder (seizures or psychosis)	4 (6.6)
Hematologic disorder	56 (91.8)
Immunologic disorder	54 (88.5)
ANA positivity	54 (88.5)
NPSLE syndromes	12 (19.7)
Aseptic meningitis	2 (3.3)
Headache	1 (1.6)
Myelopathy	1 (1.6)
Seizure disorders	4 (6.6)
Psychosis	1 (1.6)
Cranial neuropathy	1 (1.6)
Polyneuropathy	2 (3.3)
Medications	
Prednisolone	56 (91.8)
Prednisolone dose (mg/day)	7.5 (4.5–16.3)
Number of IV methylprednisolone pulse	2.0 (0.0–5.0)
Cumulative prednisolone dose (gm)	15.9 (7.4–26.9)
Hydroxychloroquine	58 (95.1)
Warfarin	7 (11.5)
Antidepressants	2 (3.3)
Anticonvulsants	0 (0.0)
Statins	17 (27.9)
Azathioprine	16 (26.2)
Mycophenolate mofetil	17 (27.9)
Calcineurin inhibitors	7 (11.5)
Cyclophosphamide	7 (11.5)

### Vitamin D levels and status

The levels and status of vitamin D in patients with SLE and HCs are shown in Table 1. SLE patients were more likely than HCs to use any vitamin D supplements (93.4% versus 19.7%, p < 0.001), particularly vitamin D_2_ supplements (13.1% versus 0.0%, p < 0.001). Total 25(OH)D levels were similar using LC-MS/MS and Roche Elecsys vitamin D total assay methods and did not differ between SLE patients and HCs. There was no significant difference in 25(OH)D_3_ levels between SLE patients and HCs. The prevalence of total 25(OH)D deficiency was similar in SLE patients and HCs (8.2% versus 3.3%, p = 0.224). However, significantly more SLE patients than HCs had 25(OH)D_3_ deficiency (19.7% versus 3.3%, p = 0.003).

### Group differences in performance on ANAM testing

**Fig 1:**
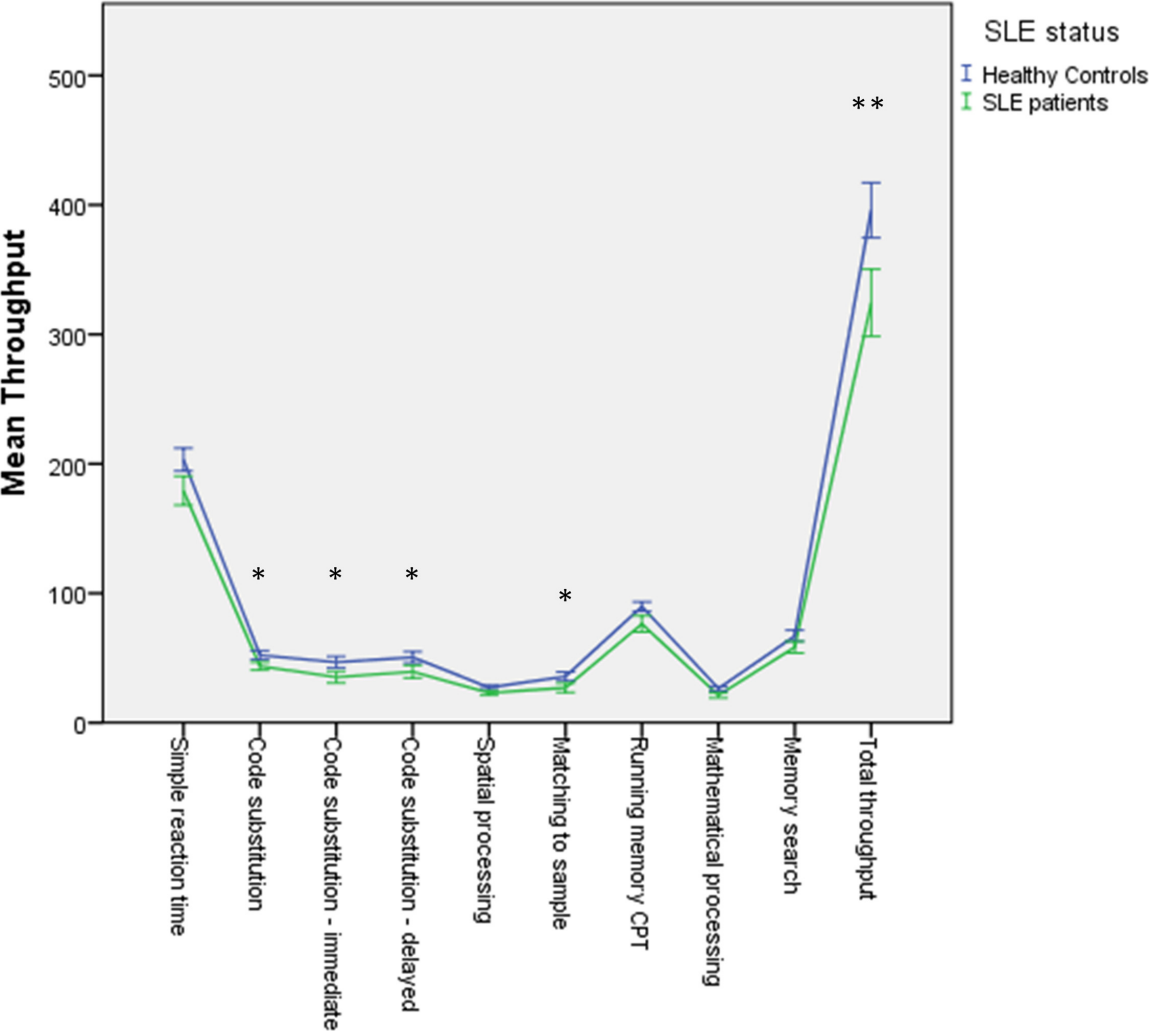
Mean throughput scores in SLE patients and HCs. Higher scores represent better performance. The individual test data points for each group are connected for illustration purposes only. *P<0.05; **P<0.01.

Throughput scores for each of the ANAM tests and TTS for SLE patients and HCs are summarized in Table 3. After adjusting for age, education, gender, ethnicity and HADS-Total, SLE patients scored significantly lower than HCs in 4 of the ANAM tests (code substitution-learning, p = 0.013; code substitution-immediate, p = 0.012; code substitution-delayed, p = 0.025; matching to sample, p = 0.026) and in the TTS (p = 0.004) (Fig 1). Simple reaction time and running memory CPT did not differ significantly between both groups. Twenty-one (34.4%) of the 61 SLE patients were identified as having cognitive dysfunction by ANAM in comparison with 1 (1.6%) of 61 HCs (p < 0.001).

**Table 3:** Comparison of ANAM throughput scores for SLE patients and healthy controls. Data are no./no. assessed (%) or mean (standard deviation).

ANAM measure	SLE patients, n = 61	Healthy controls, n = 61	p	p*
Simple reaction time	179.18 ± 43.09	203.21 ± 34.52	0.001	0.565
Code substitution (learning)	43.61 ± 11.59	52.22 ± 13.30	0.000	0.013
Code substitution (immediate memory)	34.95 ± 16.87	46.73 ± 17.15	0.000	0.012
Code substitution (delayed memory)	39.10 ± 18.72	50.61 ± 16.87	0.000	0.025
Spatial processing	22.92 ± 7.27	27.16 ± 6.49	0.001	0.098
Matching to sample	26.72 ± 13.89	35.80 ± 13.14	0.000	0.026
Running memory CPT	75.78 ± 24.66	89.70 ± 14.24	0.000	0.050
Mathematical processing	21.07 ± 7.56	26.50 ± 6.52	0.000	0.087
Memory search	58.04 ± 17.46	67.14 ± 16.70	0.004	0.108
Total throughput	322.18 ± 100.86	395.86 ± 83.26	0.000	0.004
Cognitive dysfunction**	21 (34.4)	1 (1.6)	0.000	

* Adjusted for age, education, gender, ethnicity and HADS-Total

** Cognitive dysfunction definition: A cut-off for cognitive dysfunction was defined as a total throughput score below -1.5 SD of the health controls mean.

### Vitamin D deficiency and cognitive function

**Fig 2:**
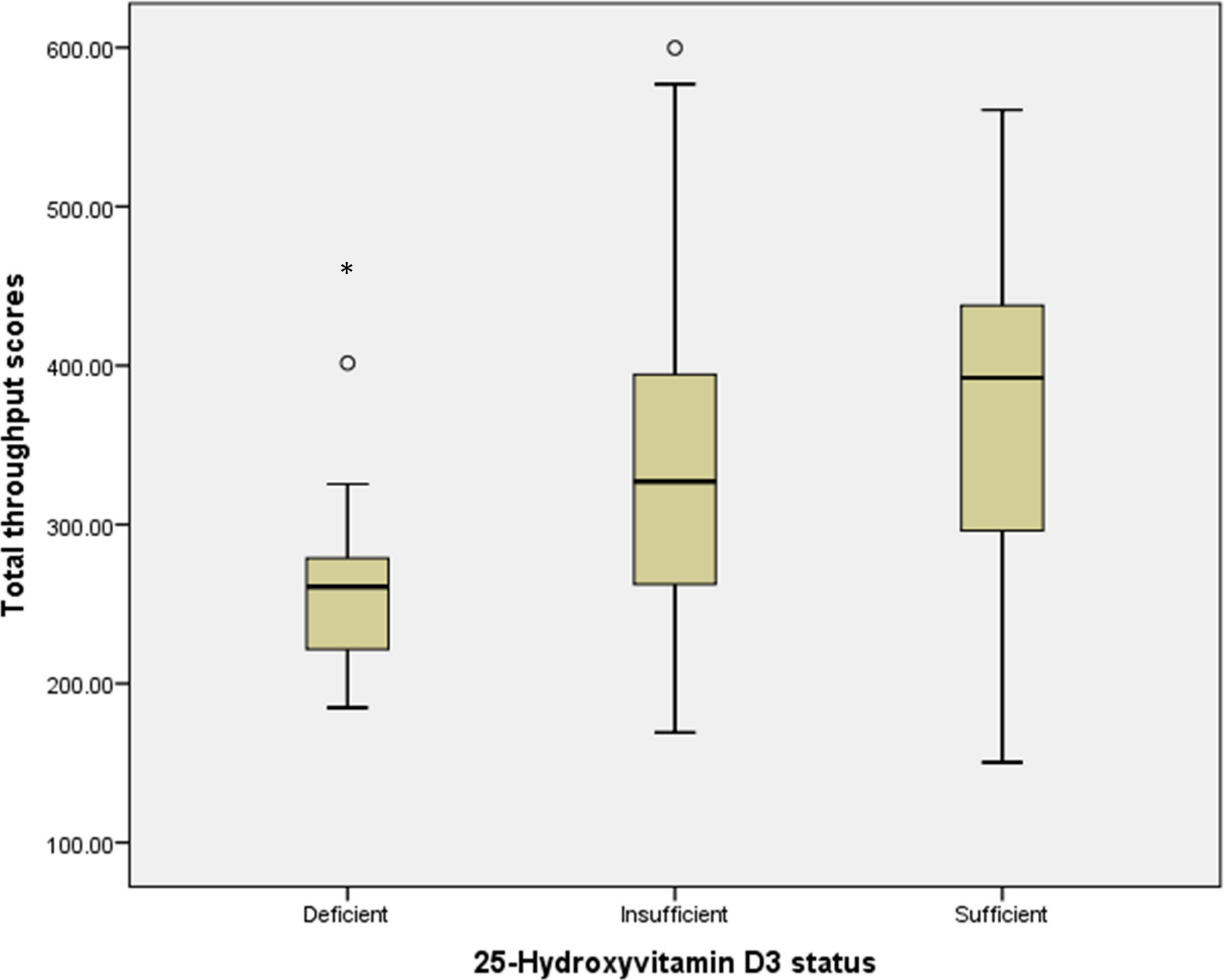
Total throughput scores in relation to 25(OH)D_3_ status in SLE patients. Box represents 25^th^ and 75^th^ percentiles, horizontal line represents the median, error bars show the 5^th^ and 95^th^ percentile and outside values are shown as dots. F(2,52) = 3.73, p = 0.031.

Multiple linear regression models were constructed for all subjects, and for HCs and SLE patients which were analyzed separately (Tables 4, 5 and 6). Age and SLE status were negative predictors for cognitive function in the regression model for all subjects, and age (β = -4.261, SE = 0.866, p < 0.001) was the only predictor for cognitive function in the HC group. Deficiency of 25(OH)D_3_, but not other vitamin D variables, independently predicted worse cognitive function in the final regression models for all subjects (β = -46.977, SE = 21.949, p = 0.035) and SLE patients (β = -63.667, SE = 27.456, p = 0.025). Mean TTS varied significantly by 25(OH)D_3_ status in SLE patients (one-way ANOVA, F(2,52) = 3.73, p = 0.031) (Fig 2). Chinese ethnicity was significantly associated with higher TTS in the SLE group. Age, education, HADS-Total and 25(OH)D_3_ levels did not differ significantly amongst the different ethnicities in the SLE group (one-way ANOVA, p > 0.05). The study sample was also analyzed for potential associations between cognitive performance and other clinical characteristics. There was no significant association between SLE patients with cognitive dysfunction and anti-dsDNA levels, stroke or antiphospholipid antibodies. Levels of 25(OH)D_3_ correlated negatively with the SLICC/ACR Damage Index (r = -0.326, p = 0.015) but not with the SELENA-SLEDAI.

**Table 4:** Results of multiple linear regression analysis between total throughput score and demographic, neuropsychological and clinical variables for 61 SLE patients and 61 healthy controls.

	Independent variable	β (SE)	Beta	P	R^2^
Model 1*					0.471
	Age	-4.523 (0.586)	-0.518	0.00	
	Chinese	59.977 (14.509)	0.285	0.00	
	SLE status (yes versus no)	-40.673 (13.778)	-0.206	0.04	
Model 2**					0.441
	Age	-4.495 (0.619)	-0.516	0.000	
	Chinese	58.515 (15.621)	0.274	0.000	
	SLE status (yes versus no)	-40.399 (14.579)	-0.205	0.007	
Model 3***					0.459
	Age	-4.422 (0.611)	-0.508	0.000	
	Chinese	53.511 (15.550)	0.250	0.001	
	SLE status (yes versus no)	-33.242 (14.732)	-0.168	0.026	
	25(OH)D_3_ deficiency	-46.977 (21.949)	-0.156	0.035	

* Model included age, education, gender, ethnicity, HADS-Total and SLE status; significant variables reported.

** Model included age, education, gender, ethnicity, HADS-Total, SLE status and 25(OH)D_3_; significant variables reported.

*** Model included age, education, gender, ethnicity, HADS-Total, SLE status and 25(OH)D_3_ status; significant variables reported.

**Table 5:** Results of multiple linear regression analysis between total throughput score and demographic, neuropsychological and clinical variables for 61 healthy controls.

	Independent variable	β (SE)	Beta	P	R^2^
Model 1*					0.300
	Age	-4.380 (0.848)	-0.558	0.00	
Model 2**					0.286
	Age	-4.261 (0.866)	-0.546	0.000	
Model 3***					0.286
	Age	-4.261 (0.866)	-0.546	0.000	

* Model included age, education, gender, ethnicity and HADS-Total; significant variable reported.

** Model included age, education, gender, ethnicity, HADS-Total and 25(OH)D_3_; significant variable reported.

*** Model included age, education, gender, ethnicity, HADS-Total and 25(OH)D_3_ status; significant variable reported.

**Table 6:** Results of multiple linear regression analysis between total throughput score and demographic, neuropsychological and clinical variables for 61 SLE patients.

	Independent variable	β (SE)	Beta	P	R^2^
Model 1*					0.452
	Age	-4.652 (0.812)	-0.548	0.000	
	Chinese	78.687 (19.200)	0.392	0.000	
Model 2**					0.420
	Age	-4.654 (0.889)	-0.543	0.000	
	Chinese	77.817 (21.248)	0.380	0.001	
Model 3***					0.462
	Age	-4.421 (0.862)	-0.516	0.000	
	Chinese	70.472 (20.712)	0.344	0.001	
	25(OH)D_3_ deficiency	-56.605 (25.047)	-0.230	0.028	
Model 4****					0.458
	Age	-4.205 (0.973)	-0.470	0.000	
	Chinese	80.290 (22.863)	0.379	0.001	
	25(OH)D_3_ deficiency	-63.667 (27.456)	-0.253	0.025	

* Model included age, education, gender, ethnicity and HADS-Total; significant variables reported.

** Model included age, education, gender, ethnicity, HADS-Total and 25(OH)D_3_; significant variables reported.

*** Model included age, education, gender, ethnicity, HADS-Total and 25(OH)D_3_ status; significant variables reported.

**** Model included age, education, gender, ethnicity, HADS-Total, 25(OH)D_3_ status, duration of SLE, SELENA-SLEDAI, SLICC/ACR Damage Index and cumulative steroid dose; significant variables reported.

## Discussion

The current study demonstrated that 25(OH)D_3_ deficiency independently predicted worse cognitive function, irrespective of the measured potential confounders. The association between cognitive function and 25(OH)D_3_ but not with total 25(OH)D is in accordance with our hypothesis that 25(OH)D_3_ represents a more accurate estimation of *in vivo* vitamin D status to affect cognitive function. Our study also confirmed that SLE patients had poorer cognitive performance on ANAM assessment compared to HCs [5, 22]. Some notable points of our study include: (i) SLE patients in the current study were not selected based on the presence and absence of NP manifestations; (ii) SLE patients had mild disease activity and disease-related damage and (iii) the absence of difference between groups in simple reaction time and running memory CPT mitigated differential effects on fatigue and inattentiveness on ANAM performance between the two groups. Thus, the inferior cognitive performance amongst the SLE patients compared to HCs in this study sample is particularly striking. While a single pattern of SLE-attributed cognitive dysfunction has not been found, commonly reported cognitive domains which are abnormal in patients with SLE include overall cognitive slowing, decreased attention, executive dysfunction and impaired working memory to suggest subcortical brain involvement reminiscent of white matter dementias; whereas, vitamin D has been associated with associated with several beneficial measures on cognitive domains associated with subcortical function, such as processing speed, attention and executive function [3, 9, 24].

Recent evidence has suggested a link between low vitamin D levels and impaired brain functioning. 25(OH)D_3_ crosses the blood-brain barrier to reach VDRs which are present on neurons and glial cells of the central nervous system (CNS), often co-localized in cells expressing 1α-hydroxylase [25, 26]. There is conversion of 25(OH)D_3_ to the biologically active 1,25(OH)_2_D_3_ in the CNS, qualifying vitamin D as a neurosteroid [27, 28]. The physiological properties of neurosteroids are diverse and differ temporally and regionally within the brain [27]. In line with this, neonatal rats exposed to vitamin D deficiency during brain development show changes in brain structure and neurochemistry [29]. 1,25(OH)_2_D_3_ has effects on neurotrophic function, neuroprotection and neuroimmunomodulation *in vitro* [10, 30]. 1,25(OH)_2_D_3_ upregulates synthesis of nerve growth factor, neurotropin 3 and low-affinity p75_NTR_ receptors [10, 30]. It regulates the intra-neuronal calcium homeostasis via voltage-gated calcium channels and exhibits neuroprotective properties against glutamate toxicity through antioxidant effects [30, 31]. Lastly, 1,25(OH)_2_D_3_ inhibits the expression of major histocompatibility complex class II proteins and sensitizes inflammatory cells to apoptotic signals [10, 30]. Functionally, the substantial expression of VDRs in the cortex and hippocampus suggests a potentially significant impact of 25(OH)D_3_ on cognition [32]. Therefore, these observations support the pharmacological potential of 25(OH)D_3_ in neuroimmunological and neurodegenerative disease management strategies [30]. Two recent meta-analyses of observational studies have concluded the potential positive effects of vitamin D in cognitive function [10, 11]. Herein, we argue that the use of vitamin D_2_ in some of the earlier studies to be study weaknesses as vitamin D_2_ administration may lead to reduction of 25(OH)D_3_ [33].

Vitamin D has a spectrum of immunoregulatory actions, many of which have been found to oppose the observed immunological aberrations in SLE [34, 35]. VDRs are expressed on immune cells of the innate and adaptive immune systems, including monocytes, macrophages,dendritic cells and lymphocytes [36]. These immune cells express 1α-hydroxylase to convert vitamin D to its biologically active form, 1,25(OH)_2_D_3_, for paracrine or autocrine signaling in the local immunologic milieu [36, 37]. 1,25(OH)_2_D_3_ binds to the VDR which acts as a transcription factor to determine a genomic response that regulates the transcription of 229 genes [38]. These VDR binding sites are significantly enriched near autoimmune genes identified from genome-wide association studies [38]. 1,25(OH)_2_D_3_ promotes chemotaxis and phagocytosis of macrophages which are important for the clearance of apoptotic cells [34]. 1,25(OH)_2_D_3_ potently inhibits type I IFN-mediated pathway of monocyte differentiation into dendritic cells [39]. By modulating the activation, maturation and survival of dendritic cells, 1,25(OH)_2_D_3_ affects the phenotype and function of interacting T cells by skewing the T cell compartment to a more anti-inflammatory and regulatory state, leading to the inhibition of Th1 and Th17 cells and induction of Th2, natural killer T and T regulatory cells [12, 40]. 1,25(OH)_2_D_3_ induces apoptosis in activated B cells and inhibits the generation of plasma cells and memory B cells [41]. Isolated peripheral blood mononuclear cells from SLE patients incubated with 1,25(OH)_2_D_3_ significantly reduced cell proliferation, as well as polyclonal and anti-dsDNA immunoglobulin production [42]. In sum, the *in vitro* data suggests a potential role of vitamin D in mitigating the pro-inflammatory pathogenic pathways of autoimmune diseases such as SLE.

Vitamin D deficiency is common in patients with SLE. It is estimated that between 8–30% of SLE patients have vitamin D deficiency during the course of the disease [12]. As Singapore lies 1° north of the equator and receives 12 hours of sunlight a day throughout the year, this study population provides a unique opportunity to evaluate vitamin D status in the absence of seasonal variation in ultraviolet B (UVB) exposure [43]. Although a single 20 min exposure of the summer sun of a fair-skinned individual can help synthesize 20,000 units of vitamin D_3_, 8.2% of SLE patients in this study were deficient in vitamin D despite supplementation [37]. This may be due to use of photo-protection, urinary loss of total 25(OH)D, medications and anti-vitamin D antibodies [35, 44]. SLE patients with active disease or cognitive dysfunction may be less inclined to spend time outdoors, as an example of reverse causality. However, most of our patients were in good health at the time of assessment with low SELENA-SELDAI scores. A recent randomized, placebo-controlled trial with vitamin D_3_ failed to diminish the interferon signature in SLE patients with total 25(OH)D levels ≤ 20 ng/mL [36]. Nonetheless, a modest improvement of SELENA-SLEDAI in the presence of higher total 25(OH)D levels has been demonstrated in SLE patients with total 25(OH)D levels < 40 ng/mL in a cohort study [45]. The larger sample size of the study by Petri *et al*. might explain why we were unable to find a relationship between 25(OH)D_3_ levels and SELENA-SLEDAI in our study [45].

More work is required to address the clinical relevance of and potential relationship between vitamin D deficiency and cognitive dysfunction in SLE patients. T2-weighted white matter hyperintensities localized to the periventricular and subcortical white matter on magnetic resonance imaging (MRI) of the brain have been described in subjects with vitamin D deficiency and SLE-attributed cognitive dysfunction [3, 46]. The clinical relevance of these T2-weighted white matter hyperintensities is related to the disruption of cortico-subcortical white matter tracts that connect brain regions important for subserving cognitive function [46]. Newly diagnosed SLE patients with no focal neurological symptoms who underwent ^18^F-fluorodeoxyglucose (^18^FDG) positron emission tomography had increased ^18^FDG uptake in the white matter, indicative of inflammatory activity [47]. This suggests that cognitive dysfunction in SLE might result from white matter inflammation as an inciting pathology with subsequent white matter tract damage [3]. The capabilities of sophisticated MRI techniques such as diffusion-tensor imaging (DTI) and spectroscopy which detect microstructural and metabolic abnormalities respectively may assist to further the understanding of hypovitaminosis D-related cognitive dysfunction in SLE [48]. The positive association with vitamin D levels and the integrity of white fibers using DTI can potentially explain our findings of higher SLICC/ACR Damage Index in SLE patients with lower 25(OH)D_3_ levels [49].

The current study has several limitations. First, the study sample was not matched for potential confounders of education and ethnicity. However, more stringent specifications would lead to a smaller sample size, but inclusion of both variables in the multiple regression models for all analyses would partially compensate for this. Second, traditional neuropsychological testing was not performed to look for clinical behavioral correlates of the abnormalities found on ANAM testing. ANAM has been validated for use in SLE to assess cognitive function and demonstrates both good sensitivity (76.2%) and specificity (82.8%) in classifying SLE patients with probable versus no impairment on traditional neuropsychological testing [18, 50]. Although ANAM does not measure complex aspects of memory, language and visuospatial functions, factor analyses have demonstrated that both ANAM and traditional neuropsychological tests measure similar underlying cognitive domains, including processing speed, working memory and resistance to interference [50, 51]. Further, ANAM provides more sensitive assessment of information processing speed and complex attention functions, which are not well assessed with traditional neuropsychological testing [18]. More importantly for this study, each ANAM test can be computer scored to yield a summary statistic, the total throughput score (TTS), to assess overall neurocognitive efficiency as a surrogate of overall cognitive impairment (i.e. brain health) of SLE patients [5, 20]. This summary statistic allowed for multiple linear regression for us to identify 25(OH)D_3_ deficiency as an independent negative predictor for TTS. On the other hand, the ACR neuropsychological battery is composed of different instruments assessing domains of simple attention, complex attention, reasoning and/or problem solving, executive functions, memory, visual-spatial processing and psychomotor speed [52]. These instruments were developed by different agencies and the scores from different instruments cannot be combined into a single summary statistic. As a result, outputs from traditional neuropsychological testing are not compatible with such analysis. The Cognitive Symptom Inventory (CSI) has been recommended as a screening tool for SLE patients suspected of having cognitive impairment in research and clinical settings; however, later studies indicated that the CSI alone is insufficient to accurately determine the likelihood of significant cognitive impairment [52, 53]. Rather, cognitive complaints reported in the CSI are influenced by the presence of anxiety and depression [53]. Lastly, although we did not have baseline levels of 25(OH)D_3_ in our subjects before study entry, a one-time assessment of total 25(OH)D levels has been shown to moderately reflect vitamin D status of individuals over an approximately 5-year period [54].

To our knowledge, this study is the first report which addressed the potential relationship between vitamin D levels and cognitive function in SLE patients. The association between 25(OH)D_3_ deficiency and cognitive impairment in SLE is novel and may provide further insights into the pathophysiological impact of vitamin D on cognitive dysfunction. The effects of the VDR likely depend on adequate concentrations of 25(OH)D_3_ as a substrate, which makes vitamin D levels or status a crucial factor in the normal functioning of the nervous and immune systems. Further prospective studies are warranted to clarify if SLE patients with 25(OH)D_3_ deficiency are more likely to experience cognitive dysfunction and whether the correction of 25(OH)D_3_ deficiency with vitamin D_3_ supplementation could improve or even prevent the process of cognitive dysfunction amongst SLE patients.
